# *TCF7L2* rs290487 C allele aberrantly enhances hepatic gluconeogenesis through allele-specific changes in transcription and chromatin binding

**DOI:** 10.18632/aging.103442

**Published:** 2020-07-10

**Authors:** Xueyou Zhang, Panpan Ye, Haitao Huang, Baohong Wang, Fengqin Dong, Qi Ling

**Affiliations:** 1 Department of Surgery, The First Affiliated Hospital, Zhejiang University School of Medicine, Hangzhou, China; 2 Eye Center, The Second Affiliated Hospital, Zhejiang University School of Medicine, Hangzhou, China; 3 Collaborative Innovation Center for Diagnosis and Treatment of Infectious Diseases, Hangzhou, China; 4 State Key Lab for Diagnosis and Treatment of Infectious Diseases, The First Affiliated Hospital, Zhejiang University School of Medicine, Hangzhou, China; 5 Department of Endocrinology and Metabolism, The First Affiliated Hospital, Zhejiang University School of Medicine, Hangzhou, China

**Keywords:** ATAC-seq, ChIP-seq, gluconeogenesis, RNA-seq, single nucleotide polymorphism, transcription factor-7-like 2

## Abstract

In this study, we investigated the mechanisms underlying the altered hepatic glucose metabolism and enhanced diabetes risk in individuals with the *TCF7L2* rs290487 C allele. Analysis of 195 cirrhotic patients revealed a higher insulin resistance index and incidence of hepatogenous diabetes in patients with the rs290487 C/C genotype compared to those with the C/T or T/T genotype. The *in vitro* experiments using targeted mutant PLC-PRF-5 cell line showed that cells with the rs290487 C/C genotype (C/C cells) had higher glucose production, lower glucose uptake, and lower *TCF7L2* mRNA and protein levels than those with the C/T genotype (C/T cells). Integrated multi-omics analysis of ChIP-seq, ATAC-seq, RNA-seq, and metabolomics data showed genome-wide alterations in the DNA binding affinity of TCF7L2 in the C/C cells, including gain (e.g., *PFKP* and *PPARGC1A*) and loss (e.g., *PGK1* and *PGM1*) of binding sites in several glucose metabolism-related genes. These allele-specific changes in transcriptional regulation lead to increased expression of gluconeogenesis-related genes (*PCK1*, *G6PC* and *PPARGC1A*) and their downstream metabolites (oxaloacetate and β-D-fructose 2,6-bisphosphate). These findings demonstrate that the *TCF7L2* rs290487 C allele enhances gluconeogenesis through allele-specific changes in transcription and chromatin binding.

## INTRODUCTION

Several intronic variants of the *transcription factor-7-like 2* (*TCF7L2*) gene are associated with increased risk of type 2 diabetes (T2D) in various ethnic groups [1–5]. The rs7903146 T allele confers the strongest risk of T2D in Caucasians by impairing β cell function [2, 3, 5]. The frequency of rs7903146 T allele is 2% among East Asians compared to 30% in the Europeans according to the HapMap data [4]. In recent decades, the prevalence of T2D has quadrupled and risen to epidemic proportions in China, but the frequency of the rs7903146 T allele remains low [1, 4, 6]. In contrast, another intronic variant rs290487 is associated with T2D risk, with a minor allele frequency of nearly 40% in the Chinese population [1]. While multiple studies have confirmed the association of rs290487 with T2D [4, 7, 8], a couple of other studies have reported no correlation [9, 10].

Functional studies suggest that the rs290487 C allele may cause insulin resistance [11–13]. Our previous study showed that rs290487 polymorphisms in the donor were associated with increased risk of new-onset diabetes in the liver transplant recipients, thereby suggesting a role for the rs290487 SNPs in the regulation of hepatic glucose metabolism [14]. In this study, we investigated the relevance of liver rs290487 SNP in hepatogenous diabetes (HD) risk by enrolling end-stage cirrhotic patients who were susceptible to HD. We generated a point mutant liver cell line carrying rs290487 C allele using CRISPR/Cas9 gene editing and explored transcriptome, chromatin binding, and metabolome changes that alter hepatic glucose metabolism via integrated multi-omics analyses of data from Ribonucleic acid sequencing (RNA-seq), Assay for transposase-accessible chromatin using sequencing (ATAC-seq), genome-wide Chromatin Immunoprecipitation followed by sequencing (ChIP-seq), and metabolomics.

## RESULTS

### *TCF7L2* rs290487 SNP is associated with hepatogenous diabetes

We analyzed the correlation between the *TCF7L2* rs290487 SNPs and clinical parameters in 195 cirrhotic patients who were susceptible to HD. As shown in Table 1, patient characteristics such as age, gender, BMI, primary liver diseases, and comorbidities were similar in patients with different rs290487 genotypes (Table 1). However, patients with the rs290487 C/C genotype showed significantly higher levels of fasting serum insulin, HOMA-IR index, and post-prandial plasma glucose compared to those with the rs290487 T/T genotype (*P* < 0.05, Table 1). Moreover, the incidence of HD was higher in patients with the rs290487 C/C (42.1%) compared to those with rs290487 C/T (27.4%) or rs290487 T/T (24.7%) genotypes, but was not statistically significant.

**Table 1:** Patient characteristics.

	**rs290487**		
	**T/T (n=81)**	**C/T (n=95)**	**C/C (n=19)**
Age (yr)	46.4 ± 9.2	45.1 ± 9.9	49.3 ± 10.2
Male (n,%)	71 (87.7)	82 (86.3)	15 (78.9)
Cirrhosis severity			
CTP score	8.8 ± 2.2	8.6 ± 2.3	9.0 ± 2.4
MELD score	20.1 ± 7.8	20.8 ± 8.6	22.2 ± 7.1
Comorbidities history			
Upper GI bleeding (n,%)	9 (11.1)	12 (12.6)	2 (10.5)
Hepatorenal syndrome (n,%)	1 (1.2)	4 (4.2)	0 (0)
Hepatic encephalopathy (n,%)	6 (7.4)	8 (8.4)	3 (15.8)
Moderate ascites (n,%)	8 (9.9)	9 (9.5)	3 (15.7)
HBV infection status			
HbsAg positive (n,%)	80 (98.8)	93 (97.9)	19 (100)
HbcAb positive (n,%)	81 (100)	93 (97.9)	19 (100)
DNA > 10^3^ copy/ml (n,%)	19 (23.5)	25 (26.3)	5 (26.3)
Metabolic parameters			
BMI (kg/m^2^)	20.7±3.4	23.5±3.3 ^a^	22.5±3.5 ^a^
Hypertension (n,%)	4 (5.0)	3 (3.2)	0 (0)
Dyslipidemia (n,%)	3 (3.7)	4 (4.2)	0 (0)
Family history of DM (n,%)	3 (3.7)	2 (2.1)	1 (5.2)
FPG (mmol/L)	5.2 (4.4, 7.2)	5.1 (4.4, 7.4)	6.0 (4.8, 9.8)
PPG (mmol/L) ^b^	13.3 (12.1, 16.0)	14.2 (12.6, 17.2)	18.3 (14.7, 20.3) ^a^
FSI (mU/L)	12.3 (9.0, 17.0)	11.0 (8.0, 15.0)	18.0 (13.0, 26.0) ^a^
HOMA-IR	2.9 (1.9, 5.5)	2.3 (1.6, 4.8)	4.5 (2.8, 11.1) ^a^
HD (n,%)	20 (24.7)	26 (27.4)	8 (42.1)

^a^ *P* < 0.05 vs. T/T using One-way ANOVA method or Mann-Whitney U test; ^b^ PPG was evaluated at 2 hr after feeding in 54 HD patients, including those with T/T (n = 20), C/T (n = 26), and C/C (n = 8) genotypes; CTP, Child-Turcotte-Pugh; GI, gastrointestinal; MELD, model for end-stage liver disease; BMI, body mass index; FPG, fasting plasma glucose; PPG, postprandial plasma glucose; FSI, fasting serum insulin; HOMA-IR, homeostasis model assessment of insulin resistance; HD, hepatogenous diabetes;

### High expression of some liver *TCF7L2* transcripts positively correlates with HOMA-IR values in patients with the rs290487 C/C genotype

**Figure 1:**
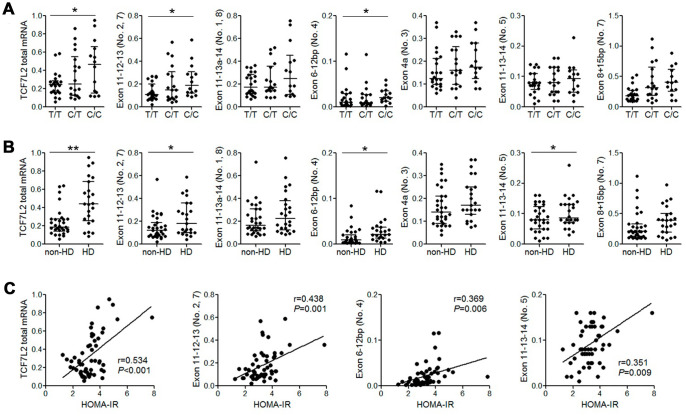
**The expression of liver TCF7L2 transcripts correlated with rs290487 variant and the diabetic status of cirrhotic patients.** (**A**) QRT-PCR analysis shows that the levels of total TCF7L2 mRNA and the liver-specific alternately spliced TCF7L2 transcripts containing exons 11-12-13 and exons 6-12bp are significantly higher in patients with the rs290487 C/C genotype compared to those with the rs290487 T/T genotype. (**B**) QRT-PCR analysis shows that levels of total TCF7L2 mRNA in the liver and liver-specific alternately spliced TCF7L2 transcripts containing exons 11-12-13, exons 6-12bp, and exons 11-13-14 were significantly higher in HD patients compared to the non-HD patients. (**C**) Correlation analysis shows that HOMA-IR value is positively associated with the levels of total TCF7L2 mRNA in the liver and the levels of liver-specific TCF7L2 transcripts. Note: *P< 0.05; ** *P*< 0.01.

Previous studies report that *TCF7L2* SNPs such as rs7903146 and rs12255372 are associated with changes in alternate-spliced *TCF7L2* transcripts and *TCF7L2* mRNA expression [15]. Therefore, we performed qRT-PCR analysis to quantify alternate *TCF7L2* transcripts in 54 patient liver tissue samples. We did not detect transcripts containing exons 11-14 (transcript No. 12) and exons 13b-14 (pancreas-specific transcript No. 9 and 10) that have been described in a previous study [16]. Moreover, patients with the rs290487 C/C variant genotype showed increased levels of total *TCF7L2* mRNA and those of liver-specific alternate-spliced *TCF7L2* transcripts such as transcript No. 2, 4, and 5 [16] compared to patients with the wild-type T/T genotype (Figure 1A). Furthermore, the levels of total *TCF7L2* mRNA and some liver transcripts were significantly higher in patients with HD than in patients without HD (Figure 1B). We also observed a positive correlation between HOMA-IR values and the levels of liver *TCF7L2* transcripts (Figure 1C).

### PLC-PRF-5 cells with the risk rs290487 C allele show enhanced hepatic gluconeogenesis

**Figure 2:**
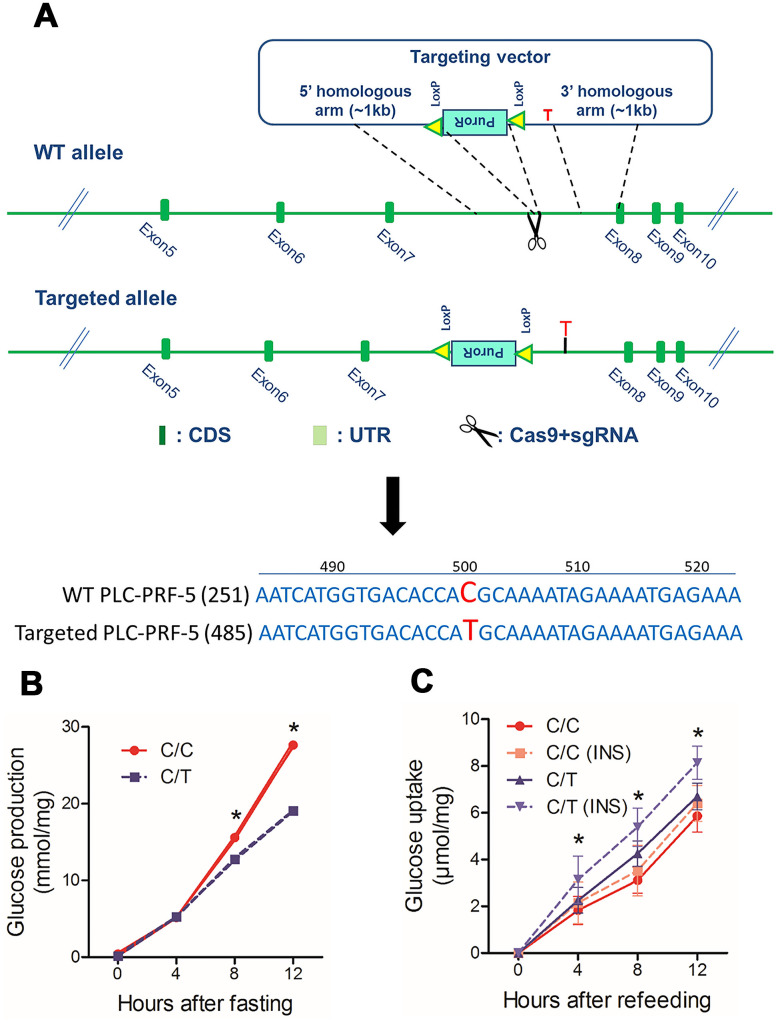
**The rs290487 variant modulates glucose metabolism in PLC-PRF-5 cells.** (**A**) Schematic diagram showing protocol for the generation of PLC-PRF-5 cell line with rs290487 C/T point mutation using the CRISPR/Cas9 knock-in approach. (**B**) Glucose production is significantly higher in C/C cells compared to the C/T cells under fasting states. (**C**) Flow cytometry results show reduced 2NBDG uptake in insulin-stimulated C/C cells compared to the C/T cells under serum-free and glucose-free conditions. *: *P*< 0.05; **: *P*< 0.01.

Next, to study the effects of rs290487 SNPs on hepatic glucose metabolism, we generated the rs290487 C/T SNP in PLC-PRF-5 cells (C/T cells) using CRISPR/Cas9 knock-in approach (Figure 2A). Targeted sequencing showed that the parental PLC-PRF-5 cells contain rs290487 C/C homozygous genotype (C/C cells). We analyzed glucose metabolism in these isogenic C/C and C/T cell lines and found increased glucose production under fasting state and decreased glucose uptake, particularly under insulin-stimulated conditions, in the C/C cells compared to the C/T cells (Figure 2B–2C).

**Figure 3:**
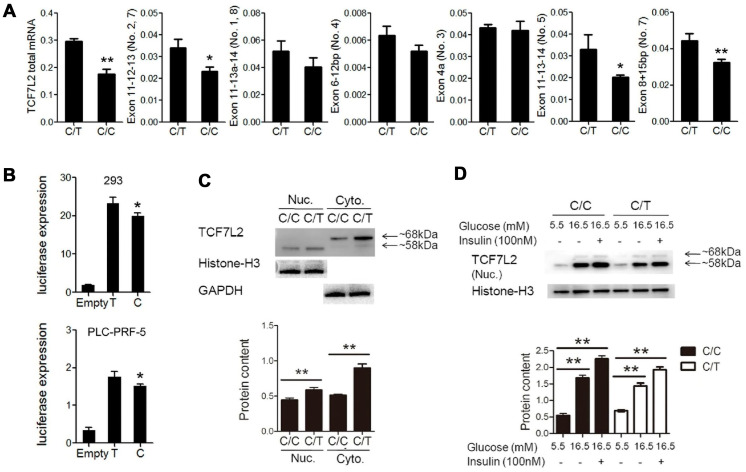
**Basic characterization of PLC-PRF-5 cells with rs290487 C/C and rs290487C/T genotypes.** (**A**) QRT-PCR analysis results show significant reduction in the expression of total TCF7L2 total mRNA and liver-specific alternative spliced transcripts containing exons 11-12-13, exons 11-13-14, and exon 8+15bp in the C/C cells compared to the C/T cells. (**B**) Dual luciferase reporter assay results show reduction in relative luciferase activity in cells transfected with pGL3-basic-promoter-rs290487 C/C transfected with pGL3-basic-promoter-rs290487 T/T. (**C**) Western blot results show that TCF7L2 protein levels are reduced in C/C cells compared to the C/T cells. (**D**) Western blot results show that TCF7L2 protein levels are increased in insulin- and high glucose- treated C/C and C/T cells. Note: * *P*< 0.05; ** *P*< 0.01.

QRT-PCR analysis showed significant reduction in the expression of total *TCF7L2* mRNA and transcript No. 5 and 7 in C/C cells compared to the C/T cells (Figure 3A). The dual luciferase reporter assays showed that the relative luciferase activity was significantly reduced in cells with the rs290487 C allele compared to those with the rs290487 T allele (Figure 3B). Western blot analysis showed that both cytoplasmic (68kDa) and nuclear (58kDa) TCF7L2 protein levels were significantly reduced in C/C cells compared to the C/T cells (Figure 3C). The distribution of the two TCF7L2 protein isoforms was confirmed in other cell lines and human liver tissues (Supplementary Figure 1).

TCF7L2 protein expression is influenced by genetic factors such as SNPs and environmental factors such as insulin and glucose [17]. We observed that treatment with insulin and high glucose concentration significantly increased TCF7L2 nuclear protein levels in both C/C and C/T cells compared to the corresponding untreated controls (Figure 3D). The effects of high glucose and insulin levels on TCF7L2 nuclear protein expression were also confirmed in several liver cell lines (Supplementary Figure 2).

### ChIP-seq and ATAC-seq show rs290487 allele-specific transcription factor binding

Next, we used ChIP-seq to determine genome-wide TCF7L2 binding sites and evaluate differential gene expression because of altered TCF7L2 protein expression.

**Figure 4:**
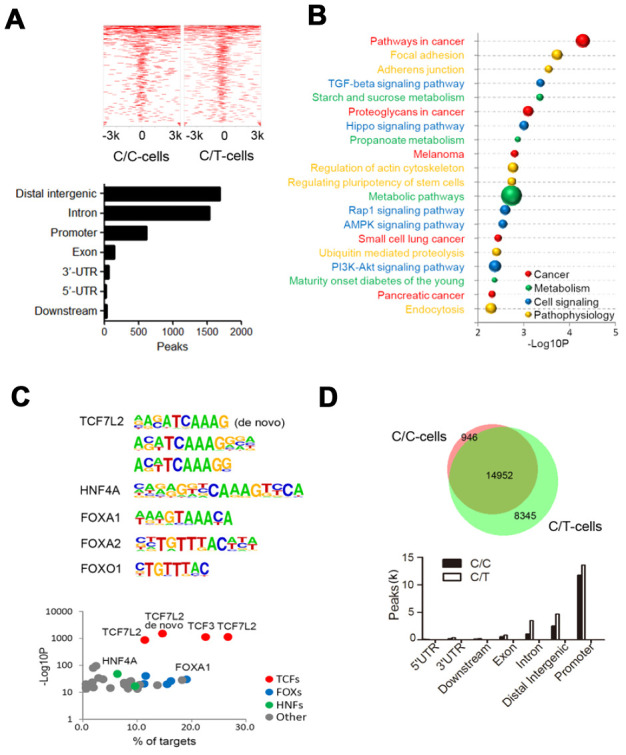
**ChIP sequencing analysis of genome-wide TCF7L2 binding specificity in PLC-PRF-5 cells with rs290487 C/C and rs290487 C/C genotypes.** (**A**) ChIP-seq analysis shows comparative genome-wide TCF7L2 binding in C/C and C/T cells. The clustered heatmap displays the distribution of TCF7L2 binding sites in a ±3 kb sequence relative to the transcript start site (TTS). Bar graph shows the genomic regions of differential ChIP-seq peaks. (**B**) The bubble chart shows the KEGG enrichment analysis of genes in the differential ChIP-seq peaks between C/C- and C/T-cells. The size of bubble represents the number of differentially expressed genes enriched in each pathway. (**C**) Differential motif enrichment analysis in the differential ChIP-seq peak regions. These regions are enriched for *de novo* and known TCF7L2 binding sites and other transcription factors that regulate gluconeogenesis. Scatter plot shows the significance of top 30 transcription factors. (**D**) ATAC-seq analysis of open chromatin regions in C/C and C/T cells. The Venn diagram represents the overlap of ATAC-seq peaks in both cells. The bar graph depicts the genomic regions in the differential ATAC-seq peaks.

We identified 7156 and 5778 peaks in C/C and C/T cells, respectively (Figure 4A). Furthermore, we identified 4101 peaks with differential TCF7L2 binding affinity (fold change > 2, *P* < 0.05), including 1934 C/C- and 1669 C/T-specific binding sites (Figure 4A). The differential binding sites were frequently in the “distal intergenic” (41.0%), “intron” (37.3%), and “promoter” (15.1%) regions of 2319 annotated genes (Figure 4A). KEGG pathway analysis of these 2319 annotated genes showed significant enrichment in cancer, metabolism, and cell signaling pathways (Figure 4B). The C/C cells showed differential binding affinity compared to C/T cells in some previously identified TCF7L2 target genes that are involved in diabetes, such as *ACSL5*, *ATM*, *AKT2*, *LEF1*, and *PDK4* [18]. Moreover, analysis of glucose metabolism-associated genes showed gain of specific TCF7L2 binding sites in genes such as *TBC1D4*, *PHKB*, *PFKP*, *PPARGC1A*, and *CREB1*, and loss of TCF7L2 binding sites in genes such as *PGK1*, *PGM1*, *PIK3CG* and *GCG*.

Then, we assessed the motifs associated with the sequences corresponding to these 4101 differential peaks and found significant enrichment in *de novo* and known TCF7L2 binding sites, thereby suggesting that the rs290487 variant altered the binding affinity of TCF7L2 protein (Figure 4C). We also found known motifs in other transcription factors such as *FOXA1*/2, *FOXO1*, and *HNF4A*, which act as TCF7L2 partners or competitors and play significant roles in modulating the expression of genes involved in gluconeogenesis (Figure 4C).

To further assess allele-specific binding, we performed genome-wide ATAC-seq. The C/C cells showed 30% fewer open chromatin regions (peaks) than the C/T cells (Figure 4D). The comparison of open chromatin structures between the C/C and C/T cells is shown in Figure 4D. There was no unique peak in the *TCF7L2* gene but weaker common peaks at intron 4 of C/C cells compared to C/T cells. Furthermore, we observed 7196 significant differential open chromatin regions (fold change > 2, *P* < 0.05) that were enriched for binding site motifs for a variety of transcription factors including TCF7L2 and its partners or competitors such as FOXA1/2, FOXO1, and HNF4A.

### Integrated analysis of RNA-seq and metabolomics reveals rs290487 allele-specific transcriptional regulation in glucose metabolic pathways

**Figure 5:**
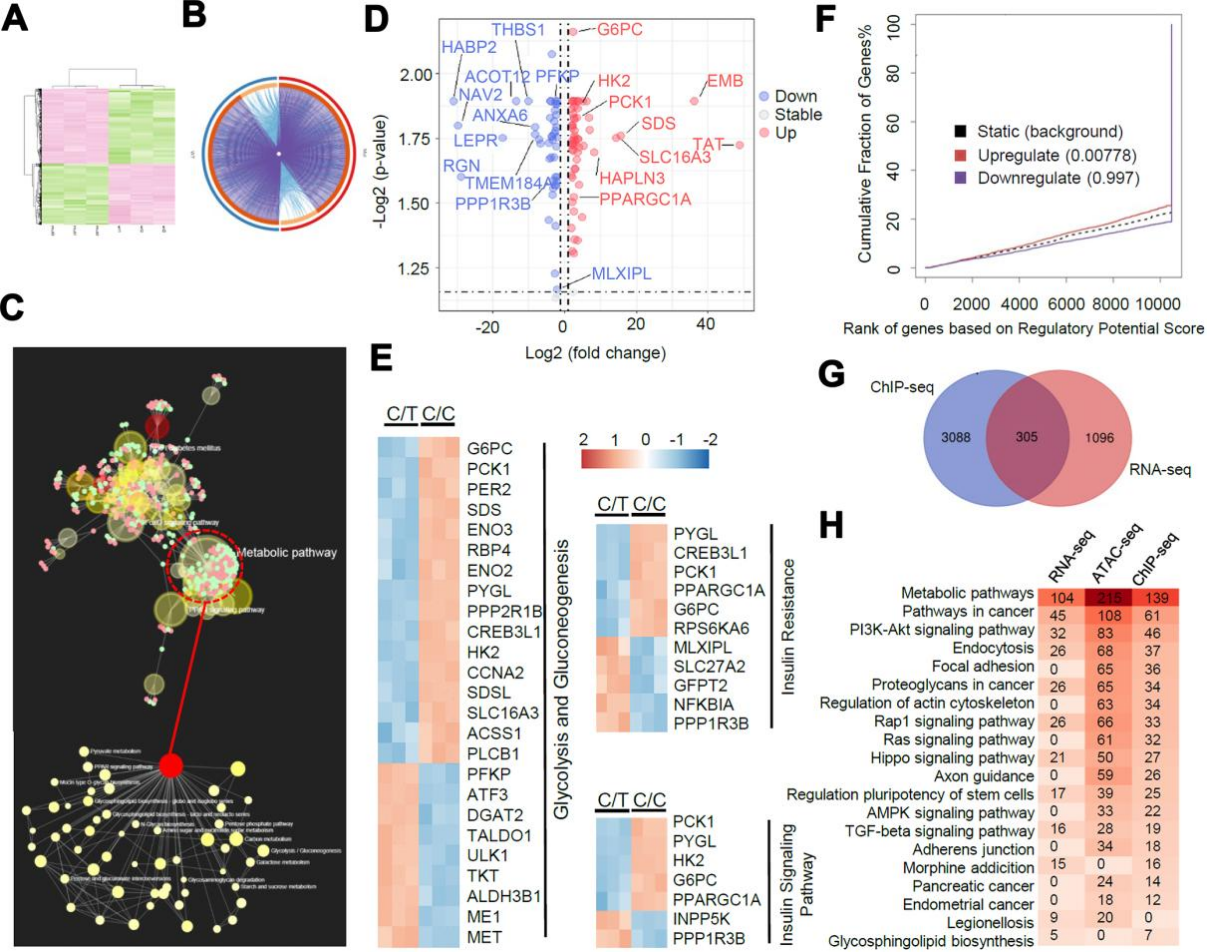
**A genome-wide analysis of transcriptome divergence in PLC-PRF-5 cells with the rs290487 C/C genotype.** (**A**) Heatmap of 1401 differentially expressed genes (DEGs) in C/C and C/T cells. (**B**) The Circos plot shows statistically significant DEGs identified by pairwise comparison of C/C vs. C/T cells. (purple lines). (**C**) KEGG enrichment analysis of 1401 DEGs using the MetaboAnalystR 2.0 server demonstrates strong enrichment in metabolic pathways (124 DEGs), particularly glucose metabolic pathways (red circle). (**D**) Scatter plot shows DEGs in glucose metabolic pathways identified by global enrichment analysis. The up- and down-regulated (fold change > 2, FDR< 0.05, *P*< 0.05) genes are annotated in red and blue, respectively. (**E**) Heatmap shows DEGs significantly enriched in glycolysis/gluconeogenesis and insulin signaling pathways. (**F**) BETA analysis shows integration of differential gene expression and TCF7L2 targets. The *P* value listed in the center represents the significance of the UP or DOWN group relative to the NON group as determined by the Kolmogorov-Smirnov test. (**G**) The diagram shows the overlap between the 1401 DEGs from the RNA-seq data analysis and the genes linked to the differential peaks from the ChIP-seq data analysis. (**H**) Integrative analysis of overlapping KEGG pathways from RNA-seq, ChIP-seq and ATAC-seq datasets. Heatmap shows the number of genes in each KEGG pathway.

We performed RNA-seq analysis to determine the allele-specific gene expression changes between C/C and C/T cells. We identified 1401 differentially expressed genes or DEGs (fold change > 2, *P* < 0.05, Figure 5A–5B). KEGG pathway analysis demonstrated that these DEGs were highly enriched in metabolic pathways (104 DEGs), especially in the glucose metabolic pathways (Figure 5C). The genes related to gluconeogenesis such as *G6PC*, *PCK1*, *PPARGC1A*, and *HNF4A* were significantly up-regulated and the genes related to insulin signaling transduction pathway such as *PPP1R3B* and *MLXIPL* were significantly down-regulated in the C/C cells compared to the C/T cells (Figure 5D–5E).

Next, we performed BETA analysis by integrating ChIP-seq and RNA-seq data to further assess the association between altered TCF7L2-DNA binding and transcription. We found that the upregulated DEGs in C/C cells were also significantly enriched in the ChIP-seq peaks, thereby indicating a transcriptional activator role for the rs290487 C variant (Figure 5F). Moreover, 305 of the 1401 DEGs were potential direct targets of altered TCF7L2 binding (Figure 5G). KEGG pathway analysis of RNA-seq, ChIP-seq, and ATAC-seq datasets showed great similarity, and were enriched in pathways associated with metabolism and cancer (Figure 5H).

**Figure 6:**
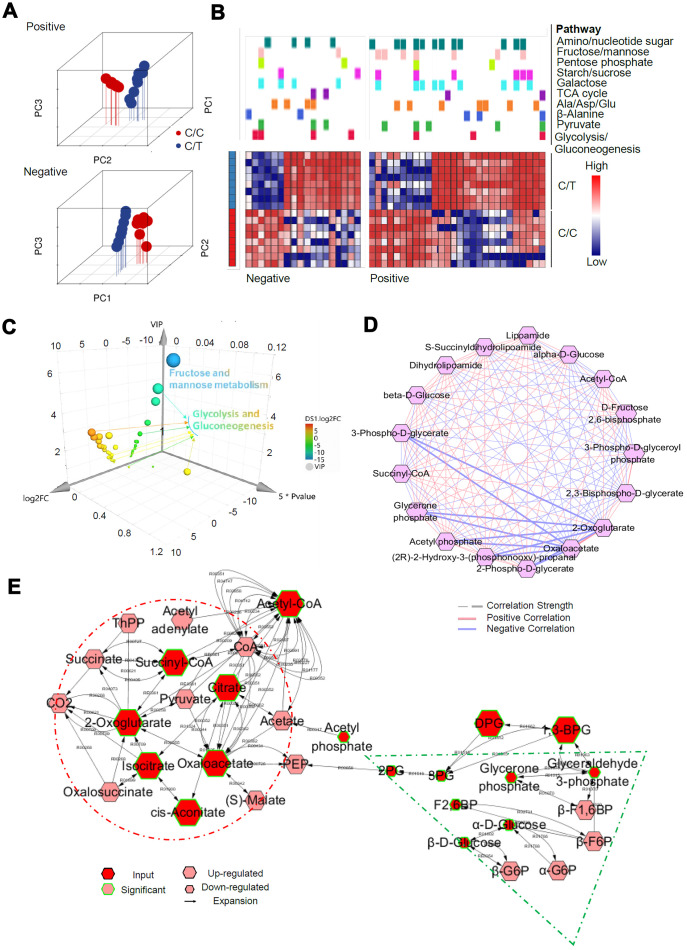
**Metabolomic alterations in PLC-PRF-5 cells with the rs290487 C/C genotype.** (**A**) 3D Principal component analysis (PCA) plots of metabolomics data from C/C and C/T cells. (**B**) Mummichog 2.0 analysis results of altered pathways (glucose metabolic pathways) in C/C cells compared to C/T cells. (**C**) 3D bubble plot shows altered metabolic features, especially changes in the glycolysis/gluconeogenesis pathway in C/C cells compared to C/T cells using SIMCA 14, the online multivariate tool. The X, Y, and Z-axis represent log_2_fold change, *P* value, and VIP, respectively. (**D**) Correlation-based network of metabolites related to glycolysis and gluconeogenesis visualized using Metscape 3. (**E**) The expression of metabolites related to glycolysis and gluconeogenesis visualized using Metscape 3. The red circle shows the increased metabolites related to gluconeogenesis such as Oxaloacetate, whereas the green triangle represents decreased glycolytic metabolites such as, D-Glyceraldehyde 3-phosphate.

We then used the UPLC-QTOF/MS based metabolomics approach to evaluate the SNP-associated metabolite profiles. Principal component analysis (PCA) was used for quality control (Figure 6A). We used the selection criteria such as Variable Importance in the Projection (VIP) > 1, fold change > 2.0, and *P*< 0.05, and identified 821/3904, and 351/1940 significant differentially expressed metabolites in positive and negative ion modes, most of which were classified as lipids, lignans, and organic acids (Supplementary Figure 3). Mummichog analysis showed that the metabolic features discriminating the C/C and C/T cell datasets were associated with glucose metabolic pathways (Figure 6B), especially glycolysis and gluconeogenesis (Figure 6C). Then, we analyzed the metabolic networks in the glycolysis and gluconeogenesis pathways (Figure 6C) and found that gluconeogenesis was increased significantly in the C/C cells (Figure 6D).

**Figure 7:**
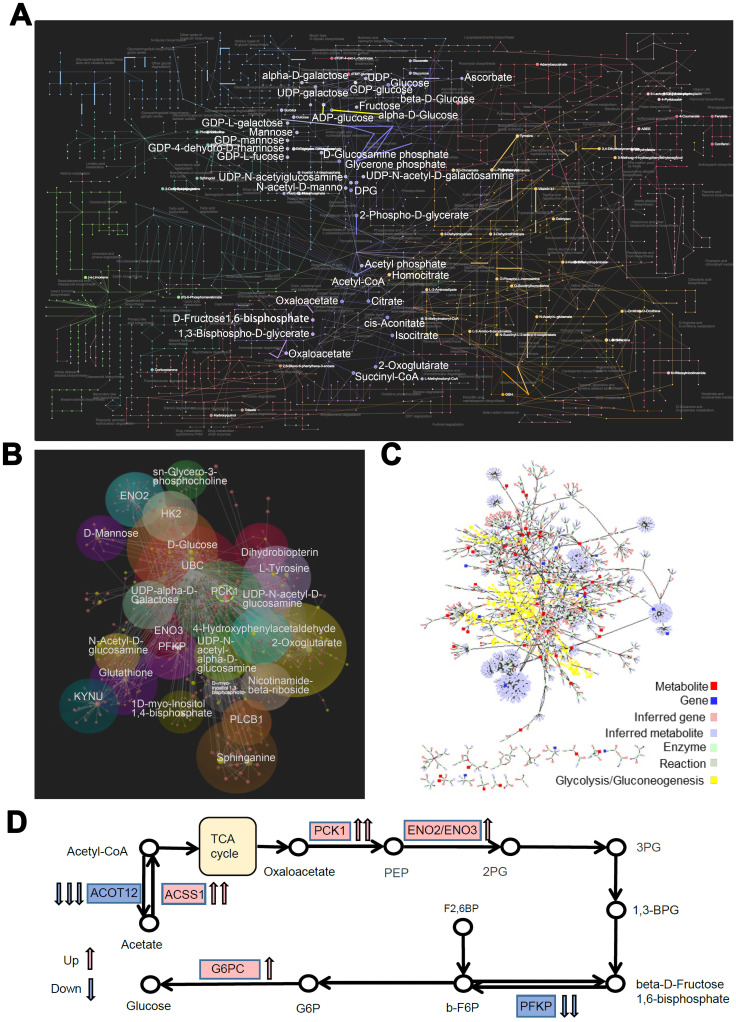
**Network and pathway analyses of transcriptome and metabolome changes in pathways related to glucose metabolism in PLC-PRF-5 cells with the rs290487 C/C genotype.** (**A**) The network shows interactions between DEGs and metabolites in the metabolic pathways visualized using OmicsNet. The bold dots and lines represent metabolites and genes in the glucose metabolic pathway, respectively. (**B**) The interactome network between transcripts and metabolites visualized using MetScape 3. The transcripts and metabolites highlighted in yellow represent those involved in the glycolysis and gluconeogenesis pathway. (**C**) The subgroup analysis of the network shows the interactions between DEGs and metabolites in the glucose metabolic pathways as visualized using OmicsNet. Red and yellow dots represent genes and metabolites, respectively. The sizes of the dots are based on their degree values. (**D**) The probable mechanism of C-allele enhanced gluconeogenesis.

Furthermore, we integrated the RNA-seq and metabolomics datasets and performed a multi-omics pathway analysis using OmicsNet [19] and Metscape 3 [20] online tools. We identified 75 significantly regulated pathways (*P* < 0.05, Supplementary Table 1) in the network, which involved interactions between 104 DEGs and 351metabolites in the metabolic pathways (Figure 7A). The top 3 metabolic pathways were endocrine resistance, glycolysis/gluconeogenesis, and amino sugar and nucleotide sugar metabolism. Further analysis of the interactome using MetScape 3 showed the importance of glucose metabolic pathways, especially glycolysis and gluconeogenesis (Figure 7B). Then we focused on the glucose metabolic pathways and constructed correlation models (subgroup of the network) between DEGs and metabolites with the same biological functions (OmicsNet, Figure 7C). *PCK1* showed the highest correlation degree within the biological network. Based on this network and pathway analyses, we propose that the rs290487 C allele enhances gluconeogenesis (Figure 7D).

## DISCUSSION

In this study, we demonstrate that *TCF7L2* rs290487 C allele significantly modulates hepatic glucose homeostasis compared to the T allele. We demonstrate that rs290487 C allele reduces the expression of liver *TCF7L2* transcripts with both short (e.g., transcript number 7) and long C-terminal (e.g., transcript number 5) as well as the corresponding small and large protein products. The smaller nuclear TCF7L2 isoform (58kDa) functions as a potent suppressor of hepatic gluconeogenesis by activating downstream target genes of the β-catenin/TCF pathway [21]. Our data demonstrates that the rs290487 C allele promotes hepatic glucose production by repressing TCF7L2 expression.

We also demonstrate genome-wide TCF7L2 rs290487-specific DNA binding. The T to C transition changes the binding affinity of TCF7L2 to known target genes [18, 22]. Moreover, T to C transition results in new TCF7L2 binding sites in genes such as *PPARGC1A* and *CREB*, as well as loss of TCF7L2 binding sites in genes such as *PGK1* and *PGM1*, all of which regulate glucose metabolism. Motif analysis indicates altered binding affinity in TCF7L2-related TFs and its competitors (e.g., FOXA1/2, FOXO1, and HNF4A), which regulate hepatic glucose metabolism. Consequently, we demonstrate allele-specific changes in the transcriptome and metabolome. Multi-omics analysis demonstrates that this rs290487 SNP specifically alters hepatic glucose metabolism, especially, glycolysis, gluconeogenesis and insulin signaling transduction. The increased expression of genes such as *PCK1*, *G6PC*, and *ENO2/3*, and their related metabolites such as oxaloacetate and 3-Phospho-D-glyceroyl phosphate shows that rs290487 C allele enhances gluconeogenesis, which consistent with the phenotype.

In comparison with the results from two previous TCF7L2 silencing studies [16, 22], our study suggests that the rs290487 C allele results in a loss-of-function phenotype. However, there are some contradictory findings that distinguish the transcription profiles of the rs290487 C allele from TCF7L2-silenced cells. For example, both TCF7L2-silencing and the rs290487 C allele increase the expression of gluconeogenesis genes such as *G6PC*, *PCK1*, and *HNF4A*. However, TCF7L2 silencing does not directly or indirectly affect *PPARGC1A* expression, as analyzed by ChIP-seq and RNA-seq [18, 22]. In contrast, the rs290487 variant increases the expression of *PPARGC1A*, probably by opening the chromatin at its promoter region (ATAC-seq) and by the presence of an additional TCF7L2 binding site (ChIP-seq). Moreover, transcriptome analysis of TCF7L2 silencing highlights a panel of transcription factors that act as transcriptional partners of TCF7L2 based on the analysis of co-enriched motifs of TCF7L2 binding sites [18]. Our study reveals that T to C transition changes the binding affinity of previously known (e.g., HNF4A, CTCF, FOXA1, HNF6, and CEBP) as well as new (e.g., FOXO1, FOXA2, and cJUN) TCF7L2 partners or competitors. This suggests that over-expression or knockdown strategies might limit the understanding of the functional role of risk TCF7L2 SNPs.

Our study also shows that environmental factors such as hormones modulate TCF7L2 expression. We demonstrate that the TCF7L2 protein levels are significantly lower in the C/C cells compared to the C/T cells. However, high glucose and insulin treatment dramatically increases the TCF7L2 protein expression in both C/C and C/T cells. These results are consistent with the findings from previous studies [16, 23]. More importantly, we demonstrate that metabolic regulation overcomes variant-related differences in the TCF7L2 protein levels. This may explain contradictory results from this study, which shows that liver *TCF7L2* mRNA levels are elevated in patients with the C/C genotype compared to those with the T/T wild type. Moreover, positive correlation between liver *TCF7L2* mRNA levels and HOMA-IR index further suggests metabolic regulation of TCF7L2 expression. Therefore, we speculate that the reason for people with risk SNPs being more susceptible to diabetes is aberrant regulation of hepatic gluconeogenesis. On the contrary, diabetes-related hyperglycemia and/or hyperinsulinemia increases TCF7L2 expression and activates β-catenin/TCF7L2 signaling [16].

Our previous study demonstrated the link between the TCF7L2 SNPs and new-onset diabetes after transplantation [14]. In this study, several genes involved cytochrome P450 mediated drug metabolism such as *CYP3A5*, *UGT1A1*, *UGT2A3*, *GSTM2* and *GSTM4* were differentially expressed between C/C and C/T cells. The analysis of differential peaks in both ChIP-seq and ATAC-seq revealed highly enriched motifs for NFAT and CREB, which are the known targets of calcineurin inhibitors that are used as immunosuppressants after solid organ transplantation [24]. We also observed significant differences in the expression of NFAT and CREB family members (e.g., NFATC3, NFATC4, CREB1, and CREB3) and the CREB binding protein in C/C and C/T cells. This suggests that rs290487 allele-specific differences may be the underlying cause for the hepatic glucose homeostasis imbalance in response to treatment with calcineurin inhibitors.

The study has several advantages and limitations. First, only cirrhotic patients who are known to have a high incidence of HD were included in the study to better elucidate the correlation between SNP and hepatic glucose metabolic disorders. However, the role of rs290487 in hepatic metabolism still needs to be investigated in patients with type 2 diabetes or healthy subjects. Second, we successfully established the point mutation model in PLC-PRF-5 cells but failed to establish it in other liver cell lines such as HepG2, SK-HEP-1, and Hep3B2.1-7 (data not shown). PLC-PRF-5 is a human hepatocarcinoma cell line whose genome contains integrated hepatitis B virus DNA and secretes virus envelope proteins [25], and is considered suitable to study liver diseases in Chinese patients. Since TCF7L2 regulates its target genes in a cell-specific manner [26], better technology is needed to construct stable models in the hepatocytes. Nevertheless, the current functional study provides significant evidence regarding the role of the rs290487 variant in the metabolic homeostasis of human liver. The conflicting results of previous studies regarding the association between SNP rs290487 and diabetes risk may be due to different sample size [1, 7–10]. We also observed smaller TCF7L2 protein (< 60kDa) as the dominant form in the nuclei of hepatocytes. Therefore, future investigations are necessary to determine the localization and functions of the smaller and larger isoforms of the TCF7L2 protein.

**Figure 8:**
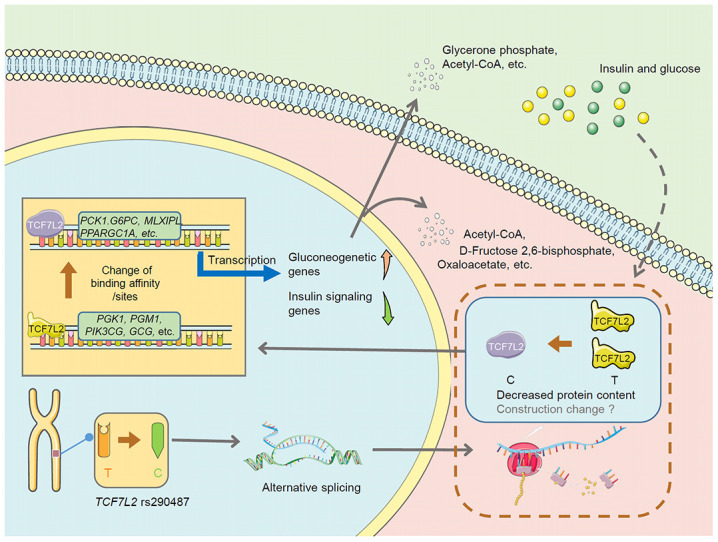
**The proposed mechanism underlying changes in hepatic glucose metabolism in the *TCF7L2* rs290487 C variant.** The *TCF7L2* rs290487 C variant shows changes in the levels of alternately spliced TCF7L2 transcripts, decreased TCF7L2 protein, and altered transcription factor-DNA binding affinity and TCF7L2 binding sites, which eventually promotes gluconeogenesis and decreases glycolysis in the rs290487 C variant when compared with the rs290487 T variant.

In summary, this is the first study to directly evaluate the effect of risk *TCF7L2* SNPs on hepatic glucose metabolism by performing multi-omics analysis of an point mutation *in vitro* model. The proposed mechanism is shown in Figure 8. Our results suggest that *TCF7L2* rs290487 SNPs may lead to changes in alternative splicing and alter protein content and structure, which subsequently changes the transcription factor-DNA binding affinity and the number of DNA-binding sites, thereby promoting gluconeogenesis. Some connections need further exploration and verification.

## MATERIALS AND METHODS

### Clinical specimens

Between 2015 and 2017, we enrolled adult hepatitis B virus-related cirrhotic patients who were admitted as liver transplant candidates to the First Affiliated Hospital, Zhejiang University School of Medicine. We excluded patients with pre-existing type diabetes mellitus 2, those in need of intensive care unit (ICU) support, or those who were not followed-up. Finally, we included 195 cirrhotic patients for this study and obtained their blood samples. We also collected 54 liver samples from abdominal surgery out of the 195 patients. The samples were immediately frozen in liquid nitrogen and stored at -80°C. We analyzed genetic polymorphisms using Applied Biosystems SNaP-Shot and TaqMan technology [14]. We obtained clinical data including age, gender, primary liver diseases, comorbidities, body mass index (BMI), and serum biochemical parameters. The diagnosis of HD and estimation of homeostatic model assessment for insulin resistance (HOMA-IR) was performed as described previously [13]. The study was in accordance with the Declaration at Helsinki, and was approved by the Ethics Committee and Institutional Review Board of The First Affiliated Hospital, Zhejiang University School of Medicine. We obtained informed consent from all patients enrolled in this study. The methodology is shown in Supplementary Figure 4.

### Cell culture, glucose production and glucose uptake tests

The PLC-PRF-5 cell line was purchased from the American Type Culture Collection (ATCC, Manassas, VA, USA) and maintained in DMEM medium supplemented with 10% fetal bovine serum. For experiments, cells were seeded in 24-well plates for 24 h. Then, we changed the medium to serum- and glucose-free DMEM medium for another 8 h. The glucose uptake and production tests have been described in our previous study [27].

### Targeted mutant PLC-PRF-5 cell line construction by the CRISPR/Cas9 system

The genome editing of PLC-PRF-5 cells was performed by the Beijing Biocytogen Co. Ltd. using the CRISPR/Cas9 system. We designed and synthesized four guide RNAs (Supplementary Table 2) and sgRNA2 to delete a 23 bp region around the SNP. We also designed cloning primers as shown in Supplementary Table 3. The *TCF7L2* mKI targeting vector was constructed by ligating the homologous arm into the LscKO-3G vector. Then we electroporated the sgRNA2 and *TCF7L2* mKI targeting vector into the PLC-PRF-5 cell line using the Celetrix CTX-1500A electroporator (Manassas, VA, US). Then, the transformed cells were selected by growing in DMEM medium containing 1μg/mL Puromycin. The positive colonies were screened by limiting dilution and genotyping using the Junction PCR (Supplementary Table 4). Eventually, we confirmed the heterozygous PLC-PRF-5 cell line with the rs290487 C/T genotype by sequencing.

### Dual-luciferase report assay

Dual-luciferase expression assay was performed as described previously [28]. The relative luciferase activity was comparable between groups. The sequence of *TCF7L2* mini promoter (1324bp) is shown in Supplementary Table 5.

### Quantitative real-time PCR

Quantitative real-time PCR was performed as described previously [28]. The qPCR primers are shown in Supplementary Table 6.

### Western blotting

Western blotting was carried out as described previously [28]. The nuclear proteins were extracted using the Nuclear and Cytoplasmic Protein Extraction Kit (Beyotime, Shanghai, China). The blots were probed with primary antibody against TCF4/TCF7L2 antibody (Cat. No. ab76151, 1:1000, Abcam, Shanghai, China). Antibodies against GAPDH (Cat. No. 60004-1-Ig, 1:5000, Proteintech, Wuhan, China) and nuclear Histone-H3 (Cat. No. 17168-1-AP, 1:3000, Proteintech) were used to detect GAPDH and Histone H3 as internal controls.

### ChIP-seq

ChIP-seq was performed by Omigen Inc. (Hangzhou, China) using the ChIP-IT Express Enzymatic shearing kit (Cat. No. 53035, Active Motif) according to manufacturer’s protocols. Briefly, the cells were harvested and fixed. Then, the cells were lysed and homogenized using the Dounce homogenizer. The cell debris was removed by centrifugation and the soluble chromatin was quantified in the Nanodrop 2000 (ThermoFisher Scientific). ChIP was performed using Dynabeads Protein G (ThermoFisher Scientific), 4 μL anti-TCF4/TCF7L2 antibody (Cat. No. 2569; Cell Signaling Tech., USA) or rabbit IgG (Cat. No. ab172730, Abcam), and 20-50 μg soluble chromatin. The beads with the protein G -antibody-chromatin complexes were washed in wash buffer according to manufacturer’s protocols. The chromatin was then reverse cross-linked by treatment with RNase A and proteinase K at 65°C overnight. The DNA was then cleaned up using the QIAGEN MinElute PCR Purification Kit (Cat. No. 28004, QIAGEN). ChIP-seq libraries were prepared using the KAPA Hyper Prep Kit (Cat. No. KK8504, Illumina) and sequenced using the Illumina HiSeq X-Ten instrument (Illumina).

### ATAC-seq

ATAC-seq was performed by Omigen Inc. Briefly, 50,000 cells from each sample were pelleted by centrifugation and lysed in buffer containing 0.1% NP40, 0.1% Tween 20, and 0.01% Digitonin to obtain the nuclei. The nuclei were then processed using the TruePrep DNA Library Prep Kit V2 for Illumina kit (Cat. No. TD501-01, Illumina) according to the manufacturer’s protocols. Immediately after transposition, the DNA fragments were purified using the MinElute PCR Purification Kit (Cat. No. 28004, QIAGEN) and PCR-amplified with the barcode and primers for 10-12 cycles. The resulting libraries were purified and assessed by the Qubit 3 Fluorometer (Invitrogen) and Agilent 2100 Bioanalyzer (Agilent), and sequenced using the Illumina HiSeq X-Ten instrument (Illumina).

### Chromatin and motif analysis

The clean sequenced reads from the ChIP- or ATAC-seq were aligned to human reference genome using Bowtie2 (v2.3.4.1) and the enriched chromatin regions were identified using MACS2 (v2.1.1). The peak visualization was performed using Integrative Genomics Viewer (v2.3.5). The differential peak analysis was performed using the MAnorm software (v1.2.0). The motifs were analyzed using the findMotifsGenome.pl from HOMER software (v4.10).

### RNA-seq

Transcriptome was sequenced by Omigen Inc. using the Illumina paired-end RNA-seq approach according to the standard protocol. Briefly, the mRNAs were purified from the total cellular RNA using the magnetic beads attached to the poly-dT oligo. Then, the double-stranded complementary DNAs were synthesized using random hexamer primers and M-MuLV Reverse Transcriptase (Invitrogen). Then, the cDNA library was constructed and sequenced using the Illumina HiSeq X-Ten instrument (Illumina).. The sequencing data was analyzed by first indexing and aligning the paired-end clean reads to the reference genome using the TopHat2 (v2.0.9) and Bowtie2 (v2.3.4.1) software. Then, the HTSeq (v0.6.1) software was used to count the number of reads that map to each gene. The DESeq2 R (1.10.1) software package was used to analyze differentially expressed genes. Enrichment analysis of the differentially expressed genes was performed using KOBAS 3.0 software.

### Metabolomics

The cells were rinsed with PBS, pelleted by centrifugation the cell pellets were quickly extracted using 1mL chilled MeOH/H_2_O (4:1, 140 v/v) and frozen in liquid nitrogen, and stored at -80°C. The ultra-performance liquid chromatography-mass spectrometry (UPLC/MS)-based metabolomics analysis was performed as described previously [29]. The raw data was processed using the Compound Discoverer 3.0 (CD 3.0, Thermo Fisher) and peak alignment, identification, and quantization was performed for each metabolite. The peaks were then matched with the mzCloud (https://www.mzcloud.org/) and the ChemSpider (http://www.chemspider.com/) database was used for qualitative and quantitative analysis. Functional and pathway analysis was performed using MetaboAnalystR 2.0 [30], SIMCA 14 [31] and mummichog algorithm. Multi-Omics molecular interaction analysis was perfrormed using OmicsNET [19] (http://www.omicsnet.ca) and Metscape 3 [20] (http://metscape.med.umich.edu/) web tools.

### Statistical analysis

SPSS version 13.0 software (SPSS Inc., Chicago, IL) was used for statistical analysis. *P* value < 0.05 was considered statistically significant. Quantitative variables are presented as mean ± SD or median ± interquartile range and compared using the Student’s *t* test or Mann-Whitney test. Categorical variables are presented as values (percentages) and compared using the Pearson’s χ^2^ test. Correlation analysis was performed using Pearson linear regression. The sequence data generated and/or analyzed in this study have been submitted to the GEO repository (GSE138781).

## Supplementary Material

Supplementary Figures

Supplementary Tables
