# Supplementary material for: TCF7L2 rs290487 C allele aberrantly enhances hepatic gluconeogenesis through allele-specific changes in transcription and chromatin binding
Source: Aging (Albany NY). 2020 Jul 10;12(13):13365–87. doi: 10.18632/aging.103442 (PMC7377900; doi:10.18632/aging.103442)
Supplement: Supplementary Figures [file aging-12-103442-s002..pdf]

SUPPLEMENTARY FIGURES

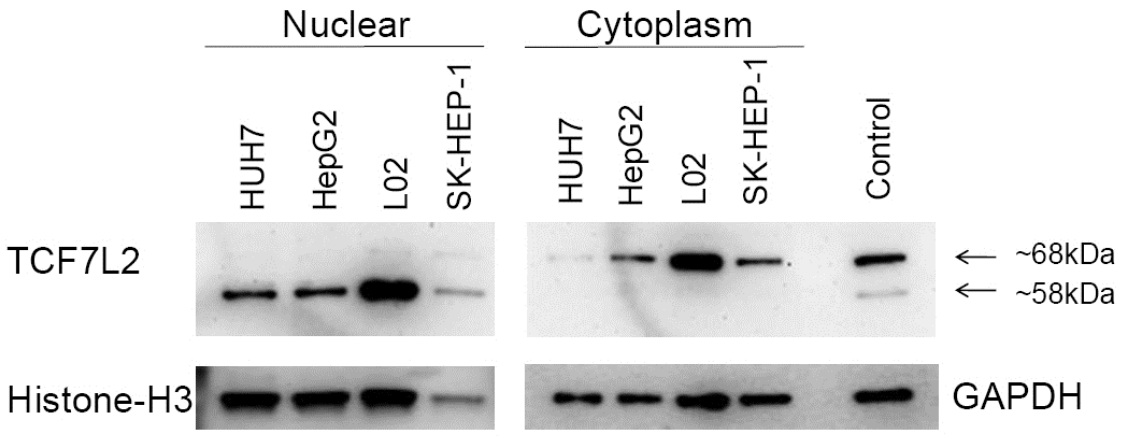

Supplementary Figure 1. TCF7L2 protein products in human liver cell lines. TCF7L2 protein products in the nuclear and cytoplasm of 4 human liver cell lines (HUH7, HepG2, Lo2, and SK-HEP-1). The total protein of PLC-PRF-5 cells was used as a positive control.

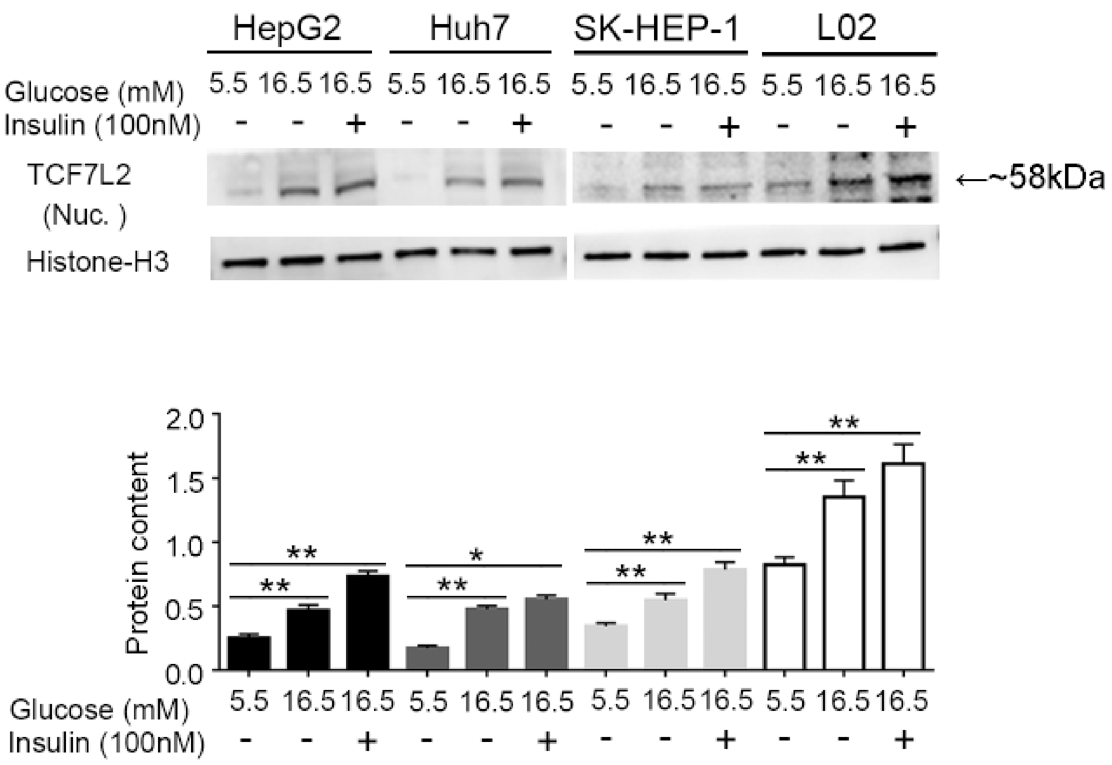

Supplementary Figure 2. TCF7L2 nuclear protein contents were increased by high glucose and insulin treatment in liver cell lines. ***P*<0.01.

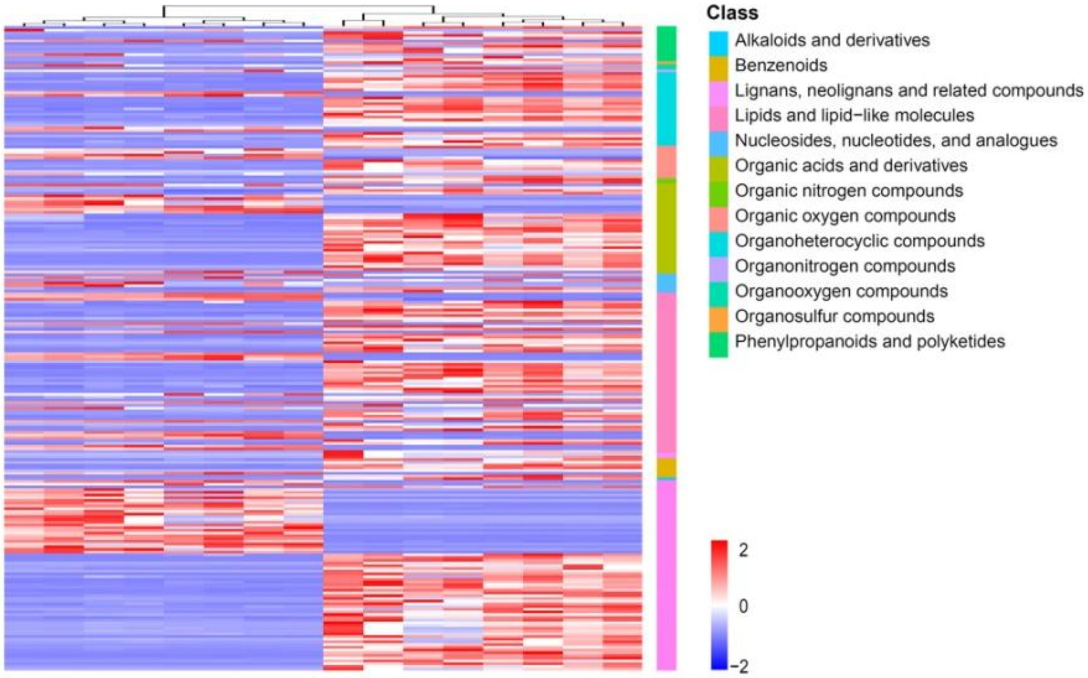

Supplementary Figure 3. Heatmap showing the classification of differential expressed metabolites (Variable Importance in the Projection >2, fold change > 5, *P*< 0.05) between C/C- and C/T-cells.

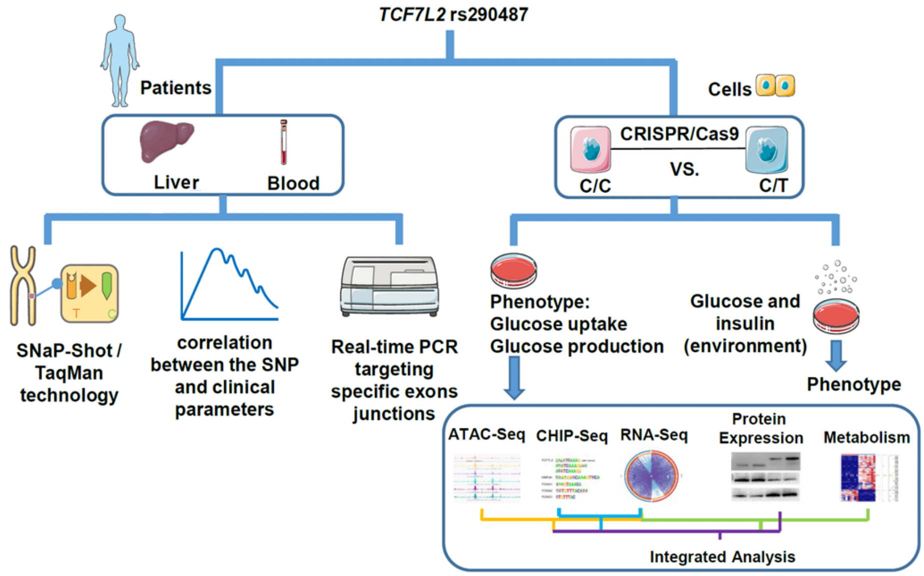

Supplementary Figure 4. A workflow showing the study design and experiments which were performed.
