# Supplementary material for: TCF7L2 rs290487 C allele aberrantly enhances hepatic gluconeogenesis through allele-specific changes in transcription and chromatin binding
Source: Aging (Albany NY). 2020 Jul 10;12(13):13365–87. doi: 10.18632/aging.103442 (PMC7377900; doi:10.18632/aging.103442)
Supplement: Supplementary Tables [file aging-12-103442-s001..pdf]

SUPPLEMENTARY TABLES

Supplementary Table 1. Significantly enriched pathways in the network (OmicsNet).

Pathway	Integrated P	hitsTotal
Endocrine resistance	4.87E-52	77
Glycolysis / Gluconeogenesis	8.53E-25	66
Amino sugar and nucleotide sugar metabolism	7.30E-23	54
Galactose metabolism	1.85E-21	42
Cortisol synthesis and secretion	6.13E-20	45
Drug metabolism - other enzymes	1.75E-16	35
Citrate cycle (TCA cycle)	5.89E-16	32
Fructose and mannose metabolism	2.68E-14	36
Glutathione metabolism	1.18E-12	49
ABC transporters	1.69E-11	60
Oocyte meiosis	1.87E-11	33
Glyoxylate and dicarboxylate metabolism	5.09E-11	27
Drug metabolism - cytochrome P450	5.99E-11	27
Metabolism of xenobiotics by cytochrome P450	2.53E-10	27
Inositol phosphate metabolism	1.56E-09	42
Central carbon metabolism in cancer	2.27E-09	20
Valine, leucine and isoleucine degradation	1.38E-08	34
Pyruvate metabolism	1.52E-08	36
Starch and sucrose metabolism	1.88E-08	32
Alanine, aspartate and glutamate metabolism	2.67E-08	25
Sphingolipid metabolism	3.14E-08	36
Phenylalanine metabolism	5.58E-08	22
Glucagon signaling pathway	1.74E-07	15
Estrogen signaling pathway	1.75E-07	33
Glycerophospholipid metabolism	2.29E-07	39
Propanoate metabolism	2.33E-07	27
Pentose phosphate pathway	3.46E-07	22
Phosphonate and phosphinate metabolism	6.05E-07	6
Type I diabetes mellitus	3.58E-06	26
Phenylalanine, tyrosine and tryptophan biosynthesis	1.18E-05	10
Folate biosynthesis	1.43E-05	23
Ascorbate and aldarate metabolism	2.43E-05	14
Tyrosine metabolism	3.87E-05	25
Pyrimidine metabolism	4.65E-05	36
Ferroptosis	6.08E-05	38
Arginine biosynthesis	7.55E-05	15
Regulation of lipolysis in adipocytes	9.70E-05	18
Renin secretion	0.00018	24
Antifolate resistance	0.00021	19
Riboflavin metabolism	0.000291	5
N-Glycan biosynthesis	0.000294	26
Arachidonic acid metabolism	0.000467	15

Mineral absorption	0.000547	19
Glycosaminoglycan degradation	0.00103	7
Tryptophan metabolism	0.00114	27
Aldosterone synthesis and secretion	0.00129	15
Glycerolipid metabolism	0.00196	21
Synthesis and degradation of ketone bodies	0.00263	9
Asthma	0.00292	27
Taurine and hypotaurine metabolism	0.00344	7
HIF-1 signaling pathway	0.00506	19
Lysine degradation	0.00523	22
Butanoate metabolism	0.00612	13
Nicotinate and nicotinamide metabolism	0.00697	21
Growth hormone synthesis, secretion and action	0.00799	10
Insulin resistance	0.00808	13
Arginine and proline metabolism	0.00986	27
Choline metabolism in cancer	0.0143	7
Mannose type O-glycan biosynthesis	0.0155	6
Selenocompound metabolism	0.0159	5
Inflammatory mediator regulation of TRP channels	0.0165	20
PI3K-Akt signaling pathway	0.0171	19
Longevity regulating pathway	0.0187	14
Vibrio cholerae infection	0.0204	12
Ether lipid metabolism	0.0241	13
Proximal tubule bicarbonate reclamation	0.0284	6
Neomycin, kanamycin and gentamicin biosynthesis	0.031	8
Glycosaminoglycan biosynthesis - chondroitin sulfate / dermatan sulfate	0.0317	5
Prolactin signaling pathway	0.0331	12
beta-Alanine metabolism	0.0374	16
Aldosterone-regulated sodium reabsorption	0.04	15
Valine, leucine and isoleucine biosynthesis	0.0469	4
Purine metabolism	0.0478	39
Tuberculosis	0.0494	7
GnRH secretion	0.0498	11

Supplementary Table 2. sgRNA design for CRISPR/Cas9 gene edit.

**Guide**		**Sequence (5'-3')**
Guide1	5'Guide	TTCTCATTTTCTATTTTGCGTGG
Guide2	3'Guide	TCAAAAATCCCACGCTGACGTGG
Guide3	3'Guide	AGGAAAGGCCACGTCAGCGTGGG
Guide4	3'Guide	TGTCATGCAGTCTTCACTACTGG
**sgRNA**		**Bottom oligo (5'-3')**
TCF7L2-sgRNA1	sg1-dn	CTATTTCTAGCTCTAAAACCGCAAAATAGAAAATGAGCGGTGTTTCGTCCTTTCCA
TCF7L2-sgRNA2	sg2-dn	CTATTTCTAGCTCTAAAACCGTCAGCGTGGGATTTTTGCGGTGTTTCGTCCTTTCCA
TCF7L2-sgRNA3	sg3-dn	CTATTTCTAGCTCTAAAACACGCTGACGTGGCCTTTCCGGTGTTTCGTCCTTTCCA
TCF7L2-sgRNA4	sg4-dn	CTATTTCTAGCTCTAAAACGTAGTGAAGACTGCATGACGGTGTTTCGTCCTTTCCA

Supplementary Table 3. Cloning primer design for targeting vector construction.

**Primer**	**Sequence(5'-3')**	**Restriction enzyme**	**Tm (°C)**	**Size (bp)**	**Template**
TCF7L2-LR-F	cgatGAATTCCCACCCTGTGACCCCCTTTAGATTG	EcoRI	63	1237	PLC-PR-F5 gDNA
TCF7L2-LR-R	cgatGTCGACACGTGGCCTTTCCTGCCACTG	SalI	64		
TCF7L2-RR-F	agctGCGGCCGCCAGCGTGGGATTTTTGAGTGAAATG	NotI	60	574	PLC-PR-F5 gDNA
TCF7L2-RR-R(in)	CACCTTTCTCATTTTCTATTTTGCATGGTGTCACCATG		64		
TCF7L2-RR-F(in)	CATGGTGACACCATGCAAAATAGAAAATGAGAAAGGTG		66	1549	PLC-PR-F5 gDNA
TCF7L2-RR-R	agctAAGCTTTATACAATTTTCCCAAGCCAGAGGGG	HindIII	61		

Supplementary Table 4. Genotyping primer design.

Primer	Sequence(5'-3')	Tm (°C)	Product size (bp)
TCF7L2-WT-F	GAAGGAACAGGGTGTCACTTGGAGC	62	WT:502
TCF7L2-WT-R	GGGCCTAATGAGGATCGATCACGAC	62	
TCF7L2-L-GT-F	AAGAAAGGTTAGGGCCTCAAGGTGC	62	Mut:1857
PuΔTK-GT-F	CTCGACTGTGCCTTCTAGTTGCCAG	62	
Neo-GT-R	CAGAGGCCACTTGTGTAGCG	59	Mut:2453
TCF7L2-R-GT-R	CGCAACCCAATAAACAAGGTCGTGG	62	

Supplementary Table 5. TCF7L2 mini promoter.

Size	Sequence
1324bp (-800~512)	CTCGAG(XhoI5')acatttaaaagaatcatatatttttccttaaggatacttttaatgtttctgacatttagcatttgtttttgcataaaaagtcgttttggcatatcca<br>tcctagtgggacttaacatttcatgaaatgattcactactaataaacaaaaaagggaggagggaggcctcatgggttaatagtttctttcattgtagatgaccaggaa<br>ctttgaccagccccttcacttccccaagctctctgaaagtggagggttgatctttccttttgaccacttttgtcgcatcccgtctaagggtggctgatttcactgctgaatt<br>aaccaccaagcacccccccacccccctcccccagccaccactttctcaaatactacccttccttttccccctcccttaagacttatttctaatatttctaaagtgcactgtt<br>ttgggcctctccccatccccgcccccaagtgggctttccttccgccttcctcggctcggattcctgacttggtcgccacccccttctcctcctctcccaccccgcatt<br>gtctttctgaaaccgccccctcccggagcaagtccctgcaccctcgcccagaatcccgggctcgcacacactccgcgcaggccgctccccctgcacactcctcc<br>ctccgtctcccccggcttccccgcccctctcttcctccttctttccctcctccctctcccggcgcccgaaaggatcattgttagccgcccccgccccgcccacccc<br>ggctgtttatttatgcacacgtcactgggccggccccgccctccggcatctcattaaggcagtgtgttcctctcgccctgtcaataatctccgctcccagactactcc<br>gttcctccggatttcgatccccctttttctatctgtcaatcagcgccgcctttgaactgaaaagctctcagtctaacttcaactcactcaaatccgagcggcacgagca<br>cctcctgtatcttcggcttcccccccccctttgctctttatatctgacttcttgttgttgttggtgttttttttttttttaccccccttttttatttattattttttgcacattgatcggatc<br>cttgggaacgagagaaaaaagaaacccaaactcacgcgtgcagaagatctcccccccccttcccctcccctcctccctcttttcccctccccaggagaaaaagacc<br>cccaagcagaaaaaagttcaccttggactcgtctttttcttgcaatatttttgggggggcaaaactttttgggggtgattttttttggcttttcttcctccttcatttttcttcc<br>aaAAGCTT(HindIII3')

Supplementary Table 6. PCR primers for detecting liver transcripts.

Primer	Target transcripts	Sequence (5'->3')	Length	Tm	GC%	Self compl.	Self 3' compl.
Exon 11-13a-14	1, 8						
Forward		CGGGAGAGACCAATGATGCA	20	59.82	55.00	4.00	4.00
Reverse		CCTTGTATGTAGCGAACGCACTTT	24	61.90	45.83	4.00	2.00
Exon 11-12-13	2,7,9,10						
Forward		CCGGGAGAGACCAATGAACACA	22	62.25	54.55	4.00	0.00
Reverse		GGGCCGCACCAGTTATTCTG	20	61.38	60.00	4.00	2.00
Exon 11-14	3,6,12,13						
Forward		CCGGGAGAGACCAATGGAGAAA	22	61.74	54.55	5.00	0.00
Reverse		CAGCGACAGAGGCTGGGTCT	20	64.03	65.00	5.00	3.00
Exon 6-12bp	4,9						
Forward		CATCCGCTAGGATGGCAAG	19	58.08	57.89	6.00	0.00
Reverse		TGCCCGTCGTGTGTAGCGTA	20	63.96	60.00	3.00	2.00
Exon 12-14	4,11						
Forward		TTCACTTCCTCCGATTACAGGAGA	24	60.81	45.83	5.00	3.00
Reverse		CAGCGACAGAGGCTGGGTCT	20	64.03	65.00	5.00	3.00
Exon 13b-14	9,10						
Forward		CGGCCCTTGCAGTCTTTGAA	20	61.17	55.00	4.00	3.00
Reverse		GCAGCTGCCTTCACCTTGTATG	22	62.08	54.55	8.00	1.00
Exon 4a	3						
Forward		AAGTTGGGGAGCCCTGGTGT	20	63.59	60.00	4.00	1.00
Reverse		CGGCGTGAAGTGTTCATTGC	20	60.73	55.00	5.00	3.00
Exon 11-13-14	5						
Forward		CGGGAGAGACCAATGACCTGA	21	61.23	57.14	3.00	1.00
Reverse		CCTTGTATGTAGCGAACGCACTTT	24	61.90	45.83	4.00	2.00
Exon 8+15bp	7						
Forward		CCATGTCCAGCTTTCTGTCTTCT	23	60.56	47.83	4.00	0.00
Reverse		CCGACATCACTCTGGGACGA	20	61.31	60.00	2.00	0.00
Exon 3-5	12						
Forward		CACCGCCCGAACCTCTAACA	20	62.16	60.00	2.00	1.00
Reverse		CGGCGTGAAGTGTTCATTGC	20	60.73	55.00	5.00	3.00
